# Standard Sample Preparation for Serial Femtosecond Crystallography

**DOI:** 10.3390/biom15111488

**Published:** 2025-10-22

**Authors:** Christina Schmidt, Kristina Lorenzen, Joachim Schulz, Huijong Han

**Affiliations:** European XFEL GmbH, Holzkoppel 4, 22869 Schenefeld, Germany; christina.schmidt@xfel.eu (C.S.); joachim.schulz@xfel.eu (J.S.)

**Keywords:** serial femtosecond crystallography, microcrystals, standard samples, protocol

## Abstract

The development of serial crystallography (SX), including serial synchrotron crystallography (SSX) at synchrotron sources and serial femtosecond crystallography (SFX) at X-ray free-electron lasers (XFELs), has facilitated the collection of high-resolution diffraction data from micron-sized crystals, providing unique insights into the structures and dynamics of biomolecules at room temperature. Standard samples are essential for the commissioning of new XFEL instruments and the validation of experimental setups. In this review, we summarize currently used standard proteins and describe representative microcrystal preparation workflows for four widely adopted models, lysozyme, myoglobin, iq-mEmerald, and photoactive yellow protein (PYP), drawing on established methodologies and accumulated experience from their applications at the European XFEL. By consolidating existing knowledge and integrating protocols that have been systematically refined and optimized through our experimental efforts, this review aims to provide practical guidance for the serial crystallography community, thereby enhancing reproducibility and ensuring consistent experimental performance across facilities.

## 1. Introduction

Serial crystallography (SX) is a revolutionary technique in structural biology that has existed for more than a decade, providing insights into the structures and dynamics of biomolecules at room temperature [1,2]. Using intense ultra-short X-ray pulses, SX made the collection of diffraction data from micron-sized crystals possible, avoiding radiation damage and enabling the capture of transient states and intermediates in biological reactions.

The success of SX experiments relies heavily on the quality of the sample [3,4,5,6]. Hence, standard samples in SX research provide critical roles. First, the commissioning of new beamline devices needs well-characterized samples. By providing consistent and reproducible diffraction patterns, standard samples help in calibrating new devices and verifying their performance. Secondly, they are required for the validation of experimental setups, ensuring that all components, from sample delivery systems to data acquisition software, function correctly.

To balance availability with practical preparation requirements, all standard samples must have robust crystallization properties. Well-documented structural data must be available for the comparability of verification measurements. Several proteins have emerged as common standards in SX research due to their ability to fulfill these criteria. These proteins not only crystallize reliably but also tolerate various sample delivery methods, data collection conditions, and experimental designs. An overview listing the most commonly used standard samples in SX experiments is given in Table 1.

The following describes several standard samples widely used in SX. Each one has specific characteristics that make it particularly suitable for instrument commissioning, method validation, or the development of time-resolved experiments.

### 1.1. Lysozyme

Lysozyme has long been the reference protein in crystallography due to its reliable crystallization behavior and well-characterized structure. In serial crystallography, its practical advantages are particularly clear because lysozyme readily forms microcrystals under a wide range of conditions, and these crystals consistently yield high-quality diffraction, making them ideal for systematic testing and method development [7,8,9,10].

The compatibility of lysozyme microcrystals with various sample delivery methods, including liquid jets [1,11], high-viscosity extrusion (HVE) [8], and fixed targets [12], has made lysozyme the protein of choice for optimizing experimental configurations. Lysozyme’s reproducibility across crystallization and data collection conditions also makes it ideal for evaluating critical aspects such as radiation damage, sample consumption, and hit rates.

Altogether, its ease of crystallization, structural consistency, and experimental flexibility have made it a foundational standard in the development and refinement of serial crystallography techniques.

### 1.2. Thermolysin

Thermolysin, a thermostable metalloprotease from *Geobacillus stearothermophilus* (34.6 kDa), is widely used as a standard sample in serial crystallography, especially for establishing different sample delivery methods such as a concentric flow electrokinetic injector, nanoflow electrospin liquid jets, on-chip crystallization, acoustic injectors and novel injection matrices, and ligand-soaking experiments, amongst others [13,14,15,16,17,18].

Available as a stable, lyophilized powder, thermolysin forms microcrystals that diffract at high resolution (up to 1.78 Å) and are robust in various experimental setups. The presence of four calcium ions and a catalytic zinc ion contributes to its pronounced stability [19,20,21]. Moreover, it can be used as a surrogate model for other zinc-dependent metalloproteases such as neprilysin (NEP), sharing conserved active-site features and substrate specificity [22].

These features make thermolysin highly suitable as both a routine standard sample and a model for mechanistic and inhibitor studies.

### 1.3. Glucose Isomerase (Xylose Isomerase)

Glucose isomerase, also known as xylose isomerase, from *Streptomyces rubiginosus* (43.3 kDa), is another standard sample in serial crystallography. Large-scale industrial production and commercial availability as a purified enzyme allow for the straightforward preparation of homogeneous microcrystals, which typically diffract to around 2 Å.

The protein has two metal-binding sites for magnesium ions in its active site, which can be replaced with other divalent metals [23]. In a study byKovalesky et al. (2010) [23], Cd^2+^ and Ni^2+^ were used as alternative metals to capture different reaction points, as their binding allows sugar binding but inhibits the catalytic reaction before and after the sugar ring-opening step, respectively. This makes glucose isomerase an interesting standard sample for the establishment of novel time-resolved mixing approaches. It is also notable that the activity of the protein is either inhibited or activated upon binding with different metals, depending on their respective ionic radii [24,25].

The protein has been used to study various viscous media as injection matrices [26,27,28,29,30], to test one fixed-target sample holder [31], and for fixed-target pink-beam serial synchrotron crystallography [32]. Additionally, it has been employed to establish mixing via a liquid application method using a spit robot [33,34] and on-chip crystallization [35].

### 1.4. Proteinase K

Proteinase K from *Engyodontium album* (formerly known as *Tritirachium album*) is a model protein for subtilisin-like serine proteases, and its function is widely studied. It is a small exo- and endoprotease (29.5 kDa) and is routinely used in molecular biology during nucleic acid isolation.

The protein can be purchased as lyophilized powder and microcrystals that diffract up to ~1.8 Å can be obtained within hours.

In serial crystallography, proteinase K has contributed to testing high-speed data acquisition (e.g., kilohertz frame rates) [12], on-chip crystallization [15,35], pink-beam experiments [36,37], SIRAS (Single Isomorphous Replacement with Anomalous Scattering) phasing [38] and other innovations across both XFEL-based and synchrotron-based platforms [39,40].

It has also been used in the development of a new sample delivery methods, including the implementation of circular motion in microfluidic sample plates [41] and in the advancement of fixed-target systems [42].

### 1.5. Trypsin

Trypsin is a well-characterized serine protease most commonly sourced from porcine or bovine pancreas and is widely employed as a standard in serial crystallography. Trypsin’s structural stability and predictable enzymatic specificity have made it valuable for benchmarking in situ data collection methods [43], high-throughput ligand screening [44], and the evaluation of new droplet microfluidic device for microcrystal production [45]. It was also used for the commissioning of a high-speed piezo-driven goniometer [46].

In addition, trypsin and its variants are used as model systems for testing protease inhibitors and for structural studies relevant to drug discovery. The range of comparative data and reproducible diffraction quality strengthens trypsin’s position as a reliable reference sample for the development and standardization of serial crystallography techniques.

### 1.6. Myoglobin

Among proteins studied by serial crystallography, myoglobin holds a special place as both a scientific milestone and a versatile experimental standard. Famous as the first protein whose structure was solved at atomic resolution, it continues to serve as a bridge between classical crystallography and modern serial methods [47,48].

The heme-containing globular protein is especially valuable for time-resolved studies, as its ligand-binding and photodissociation reactions can be triggered and monitored within microcrystals [48,49]. This property has made myoglobin a key model for investigating ultrafast structural dynamics using pump-probe serial crystallography experiments, as well as for static structure determination at room temperature [50,51,52].

Microcrystallization protocols for myoglobin are well established, though crystallization behavior varies significantly between species [48,51,53]. Therefore, crystallization conditions often need to be optimized for the specific myoglobin variant used. Despite these differences, batch crystallization of myoglobin can reliably produce large quantities of microcrystals suitable for serial experiments, supporting high-throughput data collection and compatibility with diverse sample delivery methods.

Beyond its technical merits, myoglobin’s rich history and photoreactivity have made it a preferred system for demonstrating new experimental approaches and training researchers in serial crystallography. Its continued use reflects both its scientific importance and practical value in advancing structural biology.

### 1.7. GFP and Its Derivatives

Green fluorescent protein (GFP) from the jellyfish *Aequorea victoria* (~27 kDa) is widely used in molecular biology, particularly in fluorescent imaging and as an intracellular sensor [54]. Numerous engineered variants of GFP have been developed, each with distinct properties that expand its utility across various applications [55,56,57].

GFP and its derivatives are relatively hydrophobic, exhibit considerable thermostability, and crystallize readily to produce well-diffracting crystals. Among these, the reversibly photoswitchable variant rsEGFP2 has been used to study ultrafast structural changes occurring on timescales from picoseconds to milliseconds [58,59,60].

Another derivative, eGFP, has also been featured in studies describing the production and handling of microcrystals for serial crystallography [61].

Another engineered GFP variant, iq-mEmerald, was developed as an intracellular metal sensor [62]. This synthetic derivative includes a metal-binding site engineered near the chromophore by substituting three surface residues with histidine (H147, H202, and H204). Transition metals bind to this site, modulating the fluorescence in a concentration- and metal-dependent manner. Specifically, Co^2+^, Ni^2+^, and Cu^2+^ ions quench fluorescence, whereas mixing with Zn^2+^ results in increased fluorescence. Notably, copper binding induces up to 80% fluorescence quenching, with an affinity (K_a_) of approximately 0.2 µM [62]. These properties make iq-mEmerald particularly useful for visualizing mixing efficiency and studying diffusion processes in time-resolved serial crystallography. For example, it has been employed to characterize a newly designed mix-and-inject high-viscosity extruder, taking advantage of its fluorescence sensitivity to monitor real-time mixing dynamics within the nozzle [63]. In such experiments, mixing efficiency depends on multiple factors, including the viscosity of the carrier matrix, the crystal morphology, and the arrangement of protein molecules within the crystal lattice.

### 1.8. PYP

Photoactive Yellow Protein (PYP) is a well-established model system in serial crystallography, particularly for time-resolved studies [64,65,66,67,68]. Its suitability comes from a thoroughly characterized photocycle, consistent crystallization behavior, and compatibility with pump-probe experimental setups. PYP is a small (~14 kDa), water-soluble photoreceptor protein that undergoes a trans-to-cis isomerization of its covalently bound *para*-coumaric acid (*p*CA) chromophore upon blue-light excitation, initiating a series of structural intermediates spanning femtoseconds to seconds [66,69,70,71].

PYP is readily crystallized in batch, producing homogeneous microcrystals suitable for various delivery methods, including liquid jets [66,67] and fixed targets [72]. It has played a key role in advancing time-resolved serial femtosecond crystallography (TR-SFX) at XFELs, where its full photocycle has been resolved at atomic resolution (up to 1.46 Å) under room-temperature conditions [66]. These studies confirm that SFX can reveal detailed structural changes with minimal radiation damage, even at high X-ray doses.

Comparative investigations of PYP dynamics in crystalline and solution environments have provided valuable insights into how the crystal lattice can influence reaction pathways and functional mechanisms [64,66,68,73,74].

Beyond its biological significance, PYP is extensively used to develop and validate sample delivery systems, data processing pipelines, and experimental protocols in serial crystallography. Studies with PYP have substantially advanced our understanding of light-induced signal transduction and protein dynamics.

### 1.9. Thaumatin

Thaumatin from *Thaumatococcus daniellii* is one of the most widely used standard samples in serial crystallography (SX), including both synchrotrons [39,75,76] and XFELs [30,77,78,79]. Its popularity is due to its robust and reproducible crystallization, well-characterized structure, and adaptability to a wide range of sample delivery and data collection methods. Thaumatin readily forms high-quality microcrystals in batch, allowing the preparation of dense suspensions ideal for serial experiments.

Various sample delivery approaches have been tested with thaumatin, including fixed-target chips (such as polymer-based membranes and silicon chips) [76,80], liquid jets [77], and HVE [30,39]. Direct on-chip crystallization allows efficient in situ serial data collection with minimal sample handling and low background scattering, and supports ligand soaking and high-throughput screening [15,81].

Because of its reproducibility and the abundance of comparative data available, thaumatin is routinely used for beamline commissioning, validation of sample delivery systems, and testing new data collection strategies. Well-established protocols for batch microcrystallization have been successfully adapted to various delivery systems, further supporting its role as a versatile standard in serial crystallography.

### 1.10. Granulovirus

Granulovirus occlusion bodies (OBs) are a distinctive standard sample in serial crystallography, particularly valuable for experiments that test the limits of crystal size and radiation dose tolerance. These naturally occurring protein nanocrystals, produced in vivo by insect viruses such as *Cydia pomonella* granulovirus (CpGV), encapsulate the viral protein granulin within a crystalline matrix, forming highly homogeneous particles typically measuring around 100 × 100 × 300 nm [82]. Each OB contains approximately 9000 unit cells, with volumes less than 0.016 μm^3^, making them among the smallest biological crystals used in serial crystallography [83,84,85].

Their uniformity and narrow size distribution make granulovirus OBs ideal standard samples for SFX experiments. SFX data collection at XFELs has enabled the determination of granulin structure from these OBs at atomic resolution at very high X-ray doses. The use of femtosecond pulses minimizes radiation damage, allowing high-quality data collection from these sensitive nanocrystals at room temperature [83]. Due to their reproducibility, challenging size regime, and high hit rates, granulovirus OBs have become a standard sample for evaluating and comparing different serial crystallography platforms, including both XFEL-based SFX and serial electron diffraction (SerialED) [85].

In this review, we focus on four representative standard samples: lysozyme, myoglobin, iq-mEmerald, and PYP. Each was selected for its distinct characteristics and relevance to different aspects of SX experiments. Our selection criteria were based on the diverse physicochemical properties, functional relevance, and established or emerging roles of these proteins in proof-of-concept and method development studies.

Lysozyme serves as the classical standard for protein crystallography, thanks to its small size (14.3 kDa), commercial availability, and remarkable ability to crystallize under a wide range of conditions. The robustness of lysozyme makes it an ideal standard sample for testing sample delivery methods, crystallization replicability, injector compatibility, and data collection protocols [7]. In addition, its extensive literature coverage allows users to calibrate new workflows and compare performance directly across instruments and facilities.

Myoglobin was chosen as a standard sample because it combines well-established crystallization protocols with significant experimental flexibility. Its well-characterized heme cofactor and robust crystal formation allow it to serve as a reliable model for both static and time-resolved serial crystallography experiments. Importantly, myoglobin supports advanced studies of ultrafast structural changes, such as ligand photodissociation, making it highly relevant for pump-probe SFX applications [48]. Additionally, its widespread use across XFEL and synchrotron facilities provides a valuable standard for method development and cross-facility comparisons.

Iq-mEmerald is a variant of the green fluorescent protein designed for enhanced brightness and stability. As a fluorescent marker, it enables direct visualization of microcrystal suspension quality, facilitates monitoring of crystal flow during injection, and supports assessment of mixing with ligands. The use of iq-mEmerald provides a unique supporting tool for optimizing and troubleshooting sample delivery, especially in mixing injections [63].

PYP was selected to represent the class of light-activating proteins, which are highly relevant for time-resolved studies probing ultrafast structural changes. Its well-characterized photocycle and robust crystallization make it an ideal candidate to develop and validate experimental approaches for pump-probe SFX [66,69]. Its inclusion highlights specific challenges and solutions in preparing photosensitive samples and supports the evaluation of light-triggered reaction methodologies.

We present detailed protocols for the preparation of these standard samples specifically tailored for SFX experiments. Compared to SSX (serial synchrotron crystallography), SFX requires large quantities of highly uniform microcrystals, typically in the low micrometer range or smaller, to ensure efficient and reliable sample injection into the XFEL beam. Furthermore, sample homogeneity and filtering are critical to prevent clogging of injectors and to maximize data quality. The crystals must also be stable and well-suspended in compatible carrier media adapted to high-speed injection. Covering all steps from protein expression and purification (if applicable) to crystallization, these protocols provide comprehensive and reproducible preparation techniques and support the broader SFX community in ensuring high-quality, reliable data for both instrument commissioning and experimental validation.

**Table 1:** Summary of key properties for serial crystallography standard proteins, including UniProt identifiers, molecular weights, primary experimental applications, references, micro-crystallization conditions (only for proteins for which protocols are not reported in this article), and images of tertiary structure. Ligands and chromophores (colored) are being depicted as sticks or spheres (C = grey; Fe = orange; O = red; Zn = dark blue; N = navy blue; Ca = neon green; Mg = lime green) ^1^. Due to the breadth of available studies, only selected references are cited for each protein. * Protein buffer condition not reported in references. Tertiary structures display the secondary structure of one monomer, respectively.

Protein (Uniprot ID)	Size [kDa]	Major Use	References ^1^	Reported Micro-Crystallization Conditions	Tertiary Structure
PYP (P16133)	13.87	Proof of principle	[64,65,67]	This work	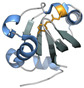 PDB ID 6P5G
		Time-resolved study	[65,66,68]		
		Sample delivery development	[72]		
Lysozyme (P00698)	14.31	Instrument commissioning	[33,86,87,88,89,90]	This work	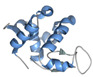 PDB ID 9I6N
		Proof of principle	[90,91]		
		Sample delivery development	[8,10,17,26,33,92,93,94]		
Myoglobin (P68082, P02185)	16.95	Instrument commissioning	[52,95,96]		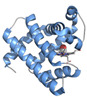 PDB ID 8BKH

		Time-resolved study	[48]	This work	
		Proof of principle	[50,52,97] [50,51]		
		Sample delivery development			
Thaumatin (P02883)	22.21	Instrument commissioning	[39]	100 mg/mL protein in ddH_2_O + 1.6 M sodium potassium tartrate [79]	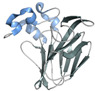 PDB ID 9FTS
		Proof of principle	[75,77,78,79]		
		Sample delivery development	[15,30,76,80,81]		
Trypsin (P00760)	23.56	Sample delivery development	[43,44,45,46]	30 mg/mL protein in 25 mM HEPES (pH 7.0), 5 mM CaCl_2_ + 100 mM Tris (pH 8.5), 30%(*w/v*) PEG 3350, 200 mM Li_2_SO_4_ [43] (30 mg/mL protein + 10 mg/mL benzamidine in 20 mM HEPES (pH 7.0), 10 mM CaCl_2_) + (20% PEG 8000, 200 mM (NH_4_)_2_SO_4_, 100 mM Bis-Tris) [44] * (65 mg/mL protein + benzamidine in 3 mM CaCl_2_) + (11–14%(*w/v*) PEG 4000, 15% ethylene glycol, 200 mM SiSO_4_, 100 mM MES (pH 6.5)) [45] (15 mg/mL protein + 5 mg/mL benzamidine in 10 mM CaCl_2_, 20 mM HEPES (pH 7.0), 3.75% PEG 3350, 5% glycerol) + (15% PEG3350, 20% glycerol) (hanging drop) [46]	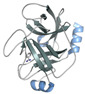 PDB ID 7WA0
iq-mEmerald	27.1	Sample delivery development	[63]	This work	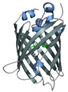 PDB ID 4KW4
rsEGFP2	26.9	Instrument commissioning	[98]	20 mg/mL protein in 2M (NH_4_)_2_SO_4_, 20 mM NaCl, 120 mM HEPEs (pH 8.0) (seeding) [59] 20–24 mg/mL protein in 75 mM HEPES (pH 8.0), 20 mM NaCl, 1.1–1.3 M (NH_4_)_2_SO_4_ (seeding) [99]	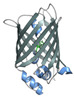 PDB ID 5O89
		Time-resolved study	[59,60,99]		
Proteinase K (P06873)	29.1	Instrument commissioning	[36,37,39,40]	40 mg/mL protein in 20 mM MES (pH 6.5) + 100 mM MES (pH 6.5), 500 mM NaNO_3_, 100 mM CaCl_2_ [39]	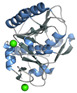 PDB ID 9FTX
		Proof of principle	[38]		
		Sample delivery development	[15,35,41,42]		
Thermolysin (P00800)	34.86	Proof of principle	[100,101]	22.5 mg/mL protein in 100 mM MES (pH 6.5) + 10 mM CaCl_2_, 5% PEG 2000 [15] * 30 mg/mL protein in 50 mM NaOH + 15% (*w/v*) ammonium sulfate [16]. * 42.5 mg/mL protein + 40% PEG 2000 MME, 0.1 M MES (pH 6.5), 5 mM CaCl_2_ [18] 330 mg/mL protein + 45% DMSO in 50 mM Tris (7.5) + 1.45 M CaCl_2_ [44]	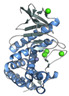 PDB ID 5WR4
		Sample delivery development	[15,16,17,18,44,46]		
Glucose isomerase (P24300)	43.33 (monomer) 173.32 (homo tetramer)	Instrument commissioning	[32]	33 mg/mL protein in 6 mM Tris (pH 7.0), 0.91 M (NH_4_)_2_SO_4_, 1 mM MgSO_4_ [31] * 80 mg/mL protein + 35%(*w/v*) PEG3350, 0.2 M LiSO4, 10 mM HEPES (pH 7.5) [35]	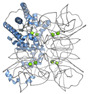 PDB ID 6KD2
		Sample delivery development	[26,27,28,29,30,31,33,34,35]		
Granulovirus (Granulin) (P87577)	29.38 (monomer) 352.56 (homo 12-mer)	Proof of principle	[83]	Not applicable	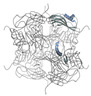 PDB ID 5G0Z
		Sample delivery development	[84]		

## 2. Materials and Methods

### 2.1. Lysozyme

#### 2.1.1. Materials

- Lysozyme: Carl Roth GmbH + Co. KG (Karlsruhe, Germany) Art.no. 8259;
- Sodium Acetate (NaOAc): Merck KGaA (Darmstadt, Germany) Art.no. 71183;
- Sodium chloride (NaCl): Carl Roth GmbH + Co. KG Art.no. P029;
- PEG 6000: Merck KGaA Art.no. 81260;
- Monoolein: Nu-Chek Prep (Elysian, MN, USA) Art.no. M-239;
- Gravity filters: CellTrics^TM^, Sysmex Deutschland GmbH (Norderstedt, Germany) (10 μm filter, Art. No. 04-0042-2314 and 20 μm filter, Art. No. 04-0042-2315);
- Gas-tight glass syringe: Hamilton Bonaduz AG (Bonaduz, Switzerland), Art.no. 202668;
- Syringe coupler:Rigaku Holdings Corporation (Tokyo, Japan), Art.no. EB-LCP-SUNION.

#### 2.1.2. Prepared Solutions

- 0.5 M NaOAc, pH 3.5;
- 5 M NaCl;
- 50% (*w/v*) PEG 6000;
- Crystallization solution: 0.1 M NaOAc, pH 3.5, 5% PEG 6000 (*w/v*), 3.2 M NaCl, 0.2 µm filtered;
- Lysozyme solution: 100 mg/mL in 50 mM NaOAc, pH 3.5, 0.2 µm filtered;
- Storage buffer: 50 mM NaOAc, pH 3.5, 1.7 M NaCl, 0.2 µm filtered.

#### 2.1.3. Crystallization

Lysozyme crystals were generated by vortexing a 1:1 ratio of the prepared lysozyme solution and crystallization solution under controlled temperature conditions. Prior to mixing, both the protein solution and the crystallization solution were equilibrated in a thermoblock. Variations in the mixing protocol, either initiating vortexing before adding the crystallization solution or adding the crystallization solution followed by immediate vortexing, led to differences in crystal size. Therefore, a standardized mixing procedure should be employed to guarantee reproducibility.

#### 2.1.4. Storage Buffer Exchange

Following crystallization, the liquid was exchanged with storage buffer. This was achieved through three rounds of centrifugation. Due to the higher viscosity of the crystallization solution, the first centrifugation was performed at 200× *g* for one minute, while the subsequent two rounds were conducted at 100× *g* for one minute each. After each centrifugation step, the supernatant was removed, and the crystal pellet was resuspended with an equal volume of fresh storage buffer.

#### 2.1.5. Crystal Filtration and Density Adjustment

To remove remaining large particles, the stored crystals were filtered using Nylon mesh gravity filters. A 10 μm filter was used for crystals smaller than 5 μm, while a 20 μm filter was employed for crystals ranging from 5 to 10 μm. The filtered crystals were allowed to settle overnight, and the sedimented volume was measured the following day. Depending on the purpose of this sample, the density (vol % of sedimented crystal) was fixed by removing or adding the storage buffer.

#### 2.1.6. Embedding in LCP

Lysozyme crystals with sizes of 5–7 μm were prepared with aqueous solutions as described above. Prior to embedding the lysozyme crystals in the lipidic cubic phase (LCP), the LCP containing the lysozyme crystal storage buffer was prepared as follows: First, the lysozyme crystal storage solution was diluted with water to 40% of its original concentration, as the undiluted solution disrupted the LCP structure and produced an opaque material. A 40% dilution was found to be the upper limit for forming a transparent LCP using this buffer composition. The diluted solution was then transferred into a gas-tight glass syringe. A second syringe was filled with melted monoolein. The volume ratio of the two solutions was fixed at 2:5 for the diluted lysozyme crystal storage solution and monoolein, respectively. The two syringes were connected using a syringe coupler, and the solutions were mixed by moving the plungers back and forth. Once the mixture became transparent, the entire sample was transferred into one syringe, and the other syringe was removed.

The filled syringe was then connected to a third syringe containing a lysozyme crystal pellet of 10% (*v/v*) relative to the prepared LCP in the other syringe. The crystals were mixed into the prepared LCP by moving the plunger until the pellet was evenly distributed throughout the syringe.

### 2.2. Myoglobin

#### 2.2.1. Materials

- Myoglobin (equine skeletal muscle): Merck KGaA Art.no. M0630;
- Ammonium sulfate ((NH_4_)_2_SO_4_): Carl Roth GmbH Art.no. 9212.1;
- Tris (Tris-(hydroxymethyl)-amino methane): Carl Roth GmbH Art.no. 5429.3;
- Sodium dithionite: Merck KGaA Art.no. 71699;
- 40 μm frit filter: JR-1100-40P, Valco Instrument Co. Inc. (Houston, TX, USA);
- PreColumn: A-355, IDEX Health & Science LLC (Rohnert Park, CA, USA);
- Luer adapter: P-642, IDEX Health & Science LLC.

#### 2.2.2. Prepared Solutions

- 4 M (NH_4_)_2_SO_4_, 0.2 µm filtered;
- 50 mM Tris buffer, pH 7.5, 0.2 µm filtered;
- 0.5 M sodium dithionite in degassed 3.3 M (NH_4_)_2_SO_4_, prepared in a glove box (GS MEGA 4).

#### 2.2.3. Crystallization

Myoglobin powder was transferred to a 5 mL centrifuge tube, and its weight was measured. Due to the volume limit of the tube, 20–30 mg of myoglobin is the optimal amount. The powder was then dissolved in 50 mM Tris buffer (pH 7.5) to achieve a final concentration of 23.6% (*w/v*). To ensure complete dissolution, the tube was vortexed thoroughly, and a brief centrifugation step was performed to collect all liquid at the bottom.

While the myoglobin solution was being vortexed, the 4 M (NH_4_)_2_SO_4_ solution was added dropwise. The total volume of (NH_4_)_2_SO_4_ solution added was 4.55 times the volume of the Tris buffer used for myoglobin dissolution. During this process, the initially transparent brown solution became cloudy, indicating the onset of nucleation. Vortexing continued for an additional 30 s, and the mixture was left undisturbed at room temperature overnight.

#### 2.2.4. Crystal Filtration

On the following day, crystal formation was inspected, and the resulting crystal slurry was filtered through a 40 μm frit filter and placed in a pre-column, which was connected to a Luer adapter and a syringe, in order to remove undesired aggregates and to disassemble clustered crystals. For liquid jet sample delivery, the crystal suspension was diluted 2-fold relative to the original crystallization volume using 3.3 M (NH_4_)_2_SO_4_.

#### 2.2.5. Deoxygenation

Prior to placing the samples into the glove box, a 3.3 M (NH_4_)_2_SO_4_ solution was degassed using a vacuum pump. The tubes containing crystals and the degassed 3.3 M (NH_4_)_2_SO_4_ were transferred into the glove box with an oxygen concentration of less than 0.5 ppm. A 0.5 M sodium dithionite solution was added to the myoglobin crystals to reach a final concentration of 3 mM, and the reduction reaction was allowed to proceed for the next 3 min. The reaction was monitored through a color change from dark brown to light red. Once the reaction was complete, sodium dithionite was removed by buffer exchange using centrifugation. The solution was centrifuged at 500× *g* for 10 min, and the supernatant was removed. The crystal pellets were then resuspended in an equal volume of degassed 3.3 M (NH_4_)_2_SO_4_ solution. This process was repeated five times to remove sodium dithionite completely.

### 2.3. Iq-mEmerald

#### 2.3.1. Materials

- Plasmid: iq-mEmerald expression vector pET17b-iq-mEmerald without using the fusion tag. The vector was purchased from Biocat GmbH (Heidelberg, Germany) using the amino acid sequence taken from the Fluorescent Protein Data Base (https://www.fpbase.org/protein/7G47U/ (accessed on 10 September 2025)) and pET17b as vector backbone.
- Recipient cell line: *Escherichia coli* BL21(DE3) (Thermo Fisher Scientific Inc. (Waltham, MA, USA), Art.no. EC0114).
- Glassware: 5 L flasks.
- Seed Beads: Jena Bioscience GmbH (Jena, Germany), Art.no. CO-501.
- Gravity filter: CellTrics™ 30 µm, Sysmex Deutschland GmbH, Art. Nr. 04-0042-2316.
- LB-medium: Carl Roth GmbH Art.no. 6673.4.
- Ampicillin: Carl Roth GmbH Art.no. K029.2.
- IPTG (isopropyl β-D-thiogalactopyranoside): Carl Roth GmbH Art.no. 2316.5.
- Tris (Tris-(hydroxymethyl)-amino methane): Carl Roth GmbH Art.no. 5429.3.
- Sodium chloride (NaCl): Carl Roth GmbH Art.no. P029.
- Ammonium sulfate ((NH_4_)_2_SO_4_): Carl Roth GmbH Art.no. 9212.1.
- Ethanol (99.9%): Merck KGaA Art.no. 1.00983.
- Hydrophobic interaction column (HIC), e.g., HiPrep Phenyl FF (High Sub) 16/10 Cytiva, Marlborough, MA, USA Art. Nr 28936545).
- 10 kDa cut-off concentrator: Merck KGaA Art.no. UFC9010.

#### 2.3.2. Equipment

- Incubation shaker: Eppendorf SE (Hamburg, Germany) New Brunswick^TM^ Innova^®^44/44R Shaker;
- FPLC: Cytiva ÄKTA pure™ chromatography system.

#### 2.3.3. Prepared Solutions

- Antibiotic: 100 mg/mL Ampicillin stock solution in ethanol;
- 1 M IPTG (isopropyl β-D-thiogalactopyranoside); 0.2 µm filtered;
- Buffer A (Lysis Buffer): 20 mM Tris, pH 7.8, 150 mM NaCl; 0.2 µm filtered;
- Buffer B (HIC start buffer): 20 mM Tris, pH 7.8, 20% (NH_4_)_2_SO_4_ saturation, 0.2 µm filtered;
- Buffer C (HIC elution buffer): 20 mM Tris, pH 7.8, 0.2 µm filtered;
- 5 M NaCl, 0.2 µm filtered;
- 70% (NH_4_)_2_SO_4_ saturated solution;
- Crystallization Buffers: 50 mM Tris, pH 8.0, 1.5–3 M (NH_4_)_2_SO_4_ concentrations in 0.1 M increments, 0.2 µm filtered.

#### 2.3.4. Expression

One colony of *Escherichia coli* BL21 (DE3) pET17b-iq-mEmerald was inoculated in 50 mL of LB medium supplemented with 100 µg/mL Ampicillin and incubated at 37 °C at 180 rpm for 16 h. The following day, the culture was diluted to an OD_600_ of 0.1 in 1 L fresh LB medium containing 100 µg/mL Ampicillin and grown at 37 °C, 180 rpm, until the OD_600_ reached 0.6. Protein expression was induced by the addition of 0.5 mM IPTG, and the culture was incubated at 18 °C for 16 h with shaking at 180 rpm. Cells were harvested by centrifugation at 8000× *g* for 15 min to 1 h at 4 °C, and pellets were stored at −20 °C until further use.

#### 2.3.5. Purification

The purification strategy was adapted from Samarkina et al. (2009) [102].

The cell pellet was resuspended in Buffer A (10 mL Buffer A per 1 g of cell pellet). The cells were lysed by sonication with 50% amplitude and 10 s on 10 s off cycles until 400 J were reached. After centrifugation of the cell lysate at 7197× *g* for 15 min at RT, the supernatant was transferred to a new tube and incubated at 65 °C for 15 min in a thermocycler or water bath. The supernatant turned cloudy and spun down for 15 min at RT and 7197× *g*. Ten milliliters of the supernatant were mixed rapidly by brief vortexing with 3 mL of 5 M NaCl and 23.3 mL of saturated (NH_4_)_2_SO_4_ (pH 7.8), respectively. Twelve milliliters of 99.9% ethanol were added instantly, and tubes were vortexed for 30 s. After that, samples were centrifuged for 7 min at RT and 3000× *g*. Iq-mEmerald is present in the organic phase, which was carefully removed and transferred to a new tube. The protein-containing fraction was diluted to 20% (NH_4_)_2_SO_4_ saturated solution in 20 mM Tris, pH 7.8. Before subjecting the sample to a HIC equilibrated with Buffer B, it was filtered through a 0.2 µm syringe filter. Iq-mEmerald was eluted over 20 column volumes of Buffer C. The fractions containing iq-mEmerald were concentrated with a 10 kDa cut-off concentrator to 50 mg/mL, flash frozen in liquid nitrogen and stored at −80 °C.

#### 2.3.6. Crystallization

After thawing, iq-mEmerald samples were filtered with a 0.2 µm filter or centrifuged at RT for 10 min at 20,817× *g*.

##### Seedstock

To prepare a large, high-concentration seedstock, 500 µL iq-mEmerald was mixed with 500 µL of 3 M (NH_4_)_2_SO_4_ and vortexed immediately for 30 s. The next day, inhomogeneous iq-mEmerald crystals appeared. Approximately 20 to 30 small glass beads were added to a 1.5 mL reaction tube. The tube was vortexed vigorously for 5 min. The slurry was cooled down to room temperature, and vortexing was repeated until no crystals were visible under a stereo microscope (total 20 min process time).

##### Needle Crystals

To grow needle crystals with a size of 1 × 1 × 12 µm, 500 µL protein solution (50 mg/mL) was mixed with 1000 µL of 2 M (NH_4_)_2_SO_4_, 50 mM Tris (pH 8.0), as well as 5 µL seedstock. The sample was vortexed for 30 s and then incubated at RT. Crystals grew overnight to their final size.

##### Cubic Crystals

To grow cubic crystals with a size of 5 × 5 × 5 µm, 500 µL protein solution (50 mg/mL) was mixed with 1000 µL of 3 M (NH_4_)_2_SO_4_, 50 mM Tris (pH 8.0), as well as 5 µL seedstock and vortexed for 30 sec. After incubation for 1 min, 500 µL of 2.5 M (NH_4_)_2_SO_4_ was added. Crystals grew overnight to their final size.

All crystal slurries were filtered with a 20 µm gravity filter and stored at RT.

#### 2.3.7. Embedding in LCP

Iq-mEmerald crystals of various sizes and shapes, as prepared above, were suitable for embedding in LCP. For this, a transparent LCP matrix was first prepared by mixing monoolein and the iq-mEmerald crystal storage buffer, 50 mM Tris (pH 8.0) and 1.5–3 M (NH_4_)_2_SO_4_, in a 7:3 volume ratio using two gas-tight glass syringes connected by a coupler. The crystal storage buffer was transferred into one gas-tight glass syringe, and a second syringe was loaded with melted monoolein. The two syringes were connected and mixed thoroughly by repeated plunger exchange until a clear, homogeneous LCP formed. The mixture was combined into one syringe.

To incorporate the crystals, a third syringe containing an iq-mEmerald crystal pellet (10% *v/v* relative to the prepared LCP) was connected to the LCP-containing syringe. The crystals were embedded by mixing with the LCP until they were evenly distributed throughout the matrix, producing a final preparation suitable for high-viscosity injection.

### 2.4. PYP (Photoactive Yellow Protein)

#### 2.4.1. Materials

- Plasmid: pET-M11 [103]. The expression vector containing the codon-optimized gene sequence for PYP was purchased from Biocat GmbH (Heidelberg, Germany) using the full PYP sequence from UniprotKB: P16113.
- Recipient cell line: *Escherichia coli* Rosetta(DE3), Merck KGaA, Art.no. 70954-3.
- Glassware: 5 L flasks.
- Seed Beads: Jena Bioscience GmbH, Art.no. CO-501.
- PD-10 Buffer exchange columns: Cytiva, Marlborough, MA, USA, Art. No. 17085101.
- Gravity filter: CellTrics™ 30 µm, Sysmex Deutschland GmbH, Art. Nr. 04-0042-2316.
- NiNTA: Thermo Fisher Scientific Art.no. A50586.
- Gravity column: Carl Roth GmbH Art. No. 1518.1.
- LB-medium: Carl Roth GmbH Art.no. 6673.4.
- Kanamycin: Carl Roth GmbH Art.no. T832.4.
- Chloramphenicol: Thermo Scientific Chemicals Art.no. B20841.22.
- HEPES (*N*-2-Hydroxyethylpiperazine-*N*’-2-ethane sulphonic acid): Carl Roth GmbH Art.no. 9105.3.
- Sodium chloride (NaCl): Carl Roth GmbH Art.no. P029.
- Imidazole: Carl Roth GmbH Art.no. 3899.3.
- Tris (Tris-(hydroxymethyl)-amino methane): Carl Roth GmbH Art.no. 5429.3.
- Tri-sodium citrate dihydrate: Carl Roth GmbH Art.no. 3580.1.
- Citric acid: Carl Roth GmbH Art.no. 7624.1.
- *p*-Coumaric acid: Merck KGaA, Art.no. C9008.
- *N,N’*-Dicyclohexylcarbodiimide (DCC): Merck KGaA, Art.no. D80002.
- *N,N*-Dimethylformamide (DMF): Merck KGaA, Art.no. 227056.
- Sodium malonate (Na-malonate): Merck KGaA, Art.no. M4795.
- Beta-mercaptoethanol: Carl Roth GmbH Art. No. 4227.3.
- Glycerol: Carl Roth GmbH Art. No. 3783.2.
- 3 kDa cut-off concentrator: Merck KGaA Art.no. UFC9003.

#### 2.4.2. Equipment

- Incubation shaker: New Brunswick^TM^ Innova^®^44/44R Shaker;
- Anion exchange column: HiPrep Q HP 16/10 Cytiva Art.no. 29018182;
- FPLC: Cytiva ÄKTA pure™ chromatography system.

#### 2.4.3. Prepared Solutions

- Kanamycin stock solution (100 mg/mL in ddH_2_O);
- Chloramphenicol stock solution (34 mg/mL in ethanol);
- Buffer A (Lysis Buffer): 20 mM HEPES, pH 7.4, 200 mM NaCl, 5 mM Imidazole, 0.2 µm filtered;
- Buffer B (Wash buffer): 20 mM HEPES, pH 7.4, 200 mM NaCl, 10 mM Imidazole, 0.2 µm filtered;
- Buffer C (Elution buffer): 20 mM HEPES, pH 7.4, 200 mM NaCl, 300 mM Imidazole, 0.2 µm filtered;
- Buffer D (TEV protease reaction buffer): 20 mM HEPES, pH 7.4, 200 mM NaCl, 0.2 µm filtered;
- Buffer E (Anion exchange start buffer): 25 mM Tris, 0.2 µm filtered;
- Buffer F (Anion exchange elution buffer): 25 mM Tris, 1 M NaCl, 0.2 µm filtered;
- Buffer G (Storage buffer): 50 mM Citrate Buffer, pH 6.0, 0.2 µm filtered;
- Buffer H (Crystallization buffer): 3.7 M sodium malonate, pH 7, 0.2 µm filtered;
- Tobacco Etch Virus (TEV) protease: prepared following the protocol from Berg et al. (2006) [104], in 50 mM Tris (pH 8.0), 200 mM NaCl, 5 mM beta-mercaptoethanol, and 10% (*v/v*) glycerol.

#### 2.4.4. Expression

The purification procedure was adapted from Schmidt et al. (2019) [105], and chromophore production was derived from Kim et al. (2013) [106].

One colony of *Escherichia coli* Rosetta(DE3) pET-M11 (Rosetta(DE3)-PYP) was inoculated in 200 mL LB (containing 50 µg/mL Kanamycin and 34 µg/mL Chloramphenicol) in a 1000 mL flask and incubated overnight at 37 °C and 160 rpm. Twelve 5 L flasks were each filled with 1 L of LB medium containing Kanamycin (50 µg/mL). Fifteen milliliters of the preculture were added to each flask and incubated for approximately 1 h 40 min at 37 °C and 160 rpm. After the OD_600_ reached 0.5, 1 mM IPTG was added, and the cells were incubated for 16 h at 18 °C and 160 rpm. The cells were harvested by centrifugation at 8000× *g* for at least 15 min at 4 °C. The cell pellet from a 1 L culture was transferred to a 50 mL Falcon tube and stored at −20 °C.

#### 2.4.5. *p*-Coumaric Anhydride (*p*CA) Synthesis

The chemicals used are hazardous or toxic, so all tasks were performed under a fume hood and appropriate PPE was worn. DCC was dissolved in 50 mL DMF to a final concentration of 1 mM (2.7 g). Separately, *p*-Coumaric acid was dissolved in 50 mL DMF to a final concentration of 0.77 mM (2.55 g). Both solutions were stirred separately at 4 °C until fully dissolved. Once dissolution was complete, the two solutions were combined and stirred overnight at 4 °C. The synthesis can be upscaled as long as the molar ratio of 1:3:1 is kept. The next day, a light-yellow solution with visible white precipitate was obtained. The solution was centrifuged at 8000× *g* for 1 h at 4 °C. The supernatant was then aliquoted to 20 mL and stored at −80 °C.

#### 2.4.6. Purification

Four cell pellets (4 L cell culture) were used per purification. The cells were resuspended in 40 mL per liter of cell culture of Buffer A. The suspension was quite viscous and slimy. The cells were lysed by sonication with 25% amplitude for 3 min (30 s on, 1 min rest), keeping the suspension on ice. The lysate was then centrifuged at 4 °C and 8000× *g* for at least 30 min. The supernatant was transferred into a beaker and stirred slowly. While the cells were being lysed, an aliquot of *p*CA (20 mL) was taken out from the freezer and centrifuged at 7000× *g* for 30 min at RT, to get rid of any remaining precipitate. Twenty milliliters of *p*CA (5 mL per 1 L of cell culture) were added *dropwise* to the cell lysate. First, some precipitate appeared, then the solution turned bright yellow. The solution was stirred gently at 50 rpm for 2 h at RT. To remove the precipitate, the solution was centrifuged at 8000× *g* for 1 h at 4 °C.

The supernatant was loaded onto a 10 mL pre-charged NiNTA resin column (equilibrated with buffer A). The column was then washed with 5 column volumes (CV) buffer A, followed by 5 CV buffer B. The bound protein was eluted with buffer C until the eluate no longer appeared yellow. To remove the imidazole, the eluate was buffer exchanged to buffer D with a PD-10 column. The protein concentration was determined by measuring the absorption at 446 nm, using the equation: conc. (mg/mL) = OD_446_/45,000 × 14,700 × dilution factor.

TEV protease was added to the purified protein solution with a 1:20 molar ratio (protease/protein), and the solution was gently shaken overnight at 4 °C.

After digestion, 1–2 mL of NiNTA resin in a gravity column was equilibrated with buffer D, and the TEV protease reaction solution was subjected to the column. The flow through, containing the cleaved protein, was collected. The column was washed with buffer D until no yellow fractions were eluted. The uncut protein was eluted using buffer C. The cleaved PYP was concentrated and buffer-exchanged to buffer E using a PD-10 column.

The protein was then further purified using an anion exchange column and an FPLC system. The column was pre-equilibrated with buffer E before the sample was applied. The protein was eluted at 10% buffer F. The absorption was monitored at 280 nm and 446 nm and the fractions containing protein, where the A_446_/A_280_ ratio was higher than two, were collected.

The protein-containing fractions were combined, and the buffer was exchanged to buffer G using a PD-10 column. The protein was further concentrated to 100 mg/mL, sterile filtered, and flash frozen in liquid nitrogen for storage at −80 °C.

#### 2.4.7. Crystallization

To produce seedstock, 12 µL PYP (100 mg/mL) was mixed with 127 µL buffer H. After combining both solutions, the mixture was stirred slowly overnight. The resulting crystallization slurry was inhomogeneous, containing big and small crystals (Figure 6). The crystals were crushed with seed beads and vigorous vortexing. The seedstock was then diluted 1:3 with buffer H.

The crystal size can be modulated by varying the volume of added seedstock. For the preparation of 2–3 µm sized crystals, 20 µL protein solution (100 mg/mL) was mixed with 86 µL buffer H (final concentration: 3 M Na-Malonate) and 0.5 µL diluted seedstock in a centrifuge tube. The mixture was instantly vortexed and incubated at RT. Crystals appeared quickly and were fully matured within 1 h. Prior to use, the crystals were filtered with a 30 µm gravity filter.

## 3. Results

### 3.1. Lysozyme

Lysozyme is a widely used standard sample in protein crystallography due to its availability, reproducibility, and excellent diffraction quality [7,8,9,10]. Lysozyme crystals serve as a benchmark for optimizing data collection strategies and refining crystallographic methodologies. This report presents an optimized protocol for lysozyme crystallization, final preparation for beamtime, and embedding in lipid cubic phase (LCP) to generate high-quality samples suitable for various applications. Additionally, the protocol enables controlled modification of the lysozyme crystals.

**Figure 1:**
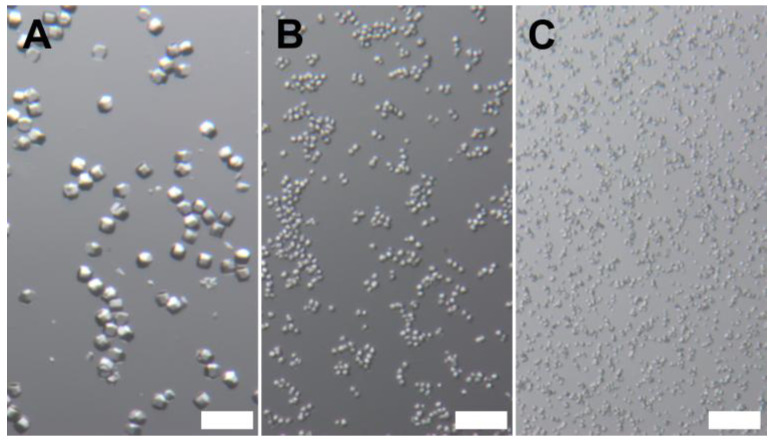
Microscope images of lysozyme crystals. (**A**) Produced at 23 °C (~14 μm); (**B**) produced at 17 °C (~5–7 μm) with vortexing first, then addition of crystallization solution; and (**C**) produced at 17 °C (~2–4 μm) with addition of crystallization solution first, then vortexing. Scale bars: 50 μm.

One important consideration is that the protocol for preparing lysozyme crystals of the desired size should be optimized whenever new solutions (lysozyme and/or crystallization solution) are prepared, as it is highly sensitive to any changes. Initial crystallization can be conducted at room temperature, with subsequent adjustments made based on the observed crystal size. As shown in Figure 1, higher temperatures (A, at 23 °C) produced larger crystals (~14 µm), while lower crystallization temperatures (B, at 17 °C) resulted in smaller crystals (~5–7 µm). The smaller crystals formed almost instantly, whereas the larger ones took up to 10 s to form. The initial optimization volume was 500 µL + 500 µL, which was later scaled up to 1.5 mL + 1.5 mL in a 5 mL centrifuge tube.

In addition, as described in the Methods section, consistency in the mixing process is critical for controlling crystal size. Figure 1B shows crystals formed by first vortexing the protein solution, followed by the addition of the crystallization solution, whereas Figure 1C shows crystals obtained when the crystallization solution was added first, followed by vortexing. As shown, the crystals in Figure 1C (~2–4 µm) are smaller than the ones in Figure 1B. This is likely due to more immediate nucleation caused by adding the crystallization solution to the protein solution while it remains still.

The final step of sample preparation for beamtime depends on the sample delivery method. One of the most common sample delivery methods at the European XFEL is the liquid jet, using GDVNs (gas dynamic virtual nozzles) or DFFNs (double-flow focus nozzles) [107,108]. In this application, a density of 15–18% (*v/v*) with 2–5 µm sized crystals provided a stable jet (Figure S1A) with a reasonable hit rate [107]. For sample delivery using HVE, the crystals were embedded in LCP, as described in the Methods section (Figure S1B, Round A. et al., in submission). This highlights the versatile use of lysozyme crystals as a standard sample for serial crystallography.

### 3.2. Myoglobin

Myoglobin crystals serve as a critical model system in serial crystallography due to their well-characterized structural properties and their ability to undergo redox reactions [48,109,110,111]. These crystals provide valuable insights into protein dynamics and ligand interactions, making them a standard sample for evaluating new methodologies in time-resolved crystallographic studies. In the Methods section, a reliable protocol is described for myoglobin crystallization, enabling the production of high-quality samples for standard applications, as well as the deoxygenation process of the crystals for time-resolved studies.

**Figure 2:**
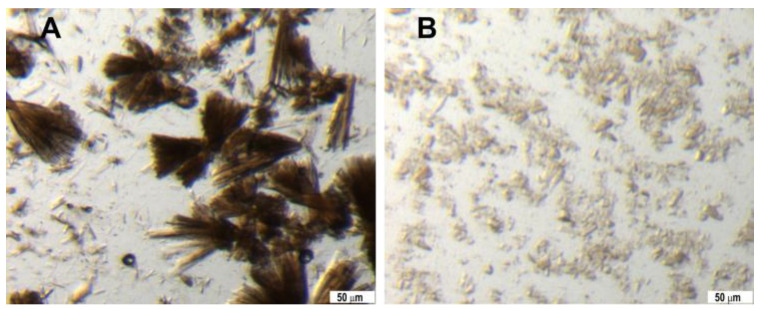
Stereomicroscope images of myoglobin crystals. (**A**) Inhomogeneous large crystals were obtained initially. (**B**) Crystals in (**A**) filtered with 40 μm filter and used for serial crystallography experiment by liquid jet. Scale bars: 50 μm.

The crystallization process outlined in the Methods section employs an unconventional ratio between the protein and precipitant (ammonium sulfate), which was determined through experimental optimization. Initially, obtaining consistent crystal growth was challenging; however, this optimized ratio reliably produced crystals. Microscopic inspection of the samples after overnight incubation revealed that some crystals formed in clusters, requiring intense filtration prior to use (Figure 2). Filtration of the crystal slurry using a 40 μm frit filter effectively removed large aggregates. Due to the lower density of myoglobin crystals compared to the crystallization solution, the crystals floated in the solution, making the estimation of crystal density difficult. Therefore, we aimed to maintain the final sample volume for the liquid jet at 2 times the original total volume used in crystallization, which resulted in a stable and reliable liquid jet (Figure S1C).

Deoxygenation of myoglobin crystals was successfully achieved using the method described here, yielding the deoxy-myoglobin state suitable for time-resolved studies of oxygen binding dynamics. In control experiments, structural analysis of deoxygenated crystals confirmed the absence of oxygen electron density at the heme site. Upon exposure to oxygen during time-resolved SFX experiments, clear electron density corresponding to bound oxygen was observed, confirming the suitability of the prepared crystals for investigating oxygen uptake mechanisms (manuscript in preparation).

The oxidized myoglobin crystals remained stable over time under ambient conditions. The optimized protocol, including crystallization, filtration, and deoxygenation steps, produced crystals suitable for serial crystallography experiments. The reproducibility of the method was confirmed through multiple independent preparations, emphasizing the strength of the protocol.

### 3.3. Iq-mEmerald

There is an ongoing need for improving sample delivery methods for serial crystallography and time-resolved experiments. As we needed a standard sample where mixing could be observed via fluorescence quenching, iq-mEmerald, a GFP derivative, was chosen [62].

The protein can be easily and quickly expressed and purified, leading to >100 mg of pure protein per liter of cell culture. Crystals of different sizes and morphologies can be produced in a reproducible manner by adjusting the concentration of the precipitant and seedstock.

One critical factor for successful mixing and pump-probe experiments is the homogeneity of the microcrystal sample. Therefore, using seedstock is most often the preferred method for producing reproducible, large batches of microcrystals.

**Figure 3:**
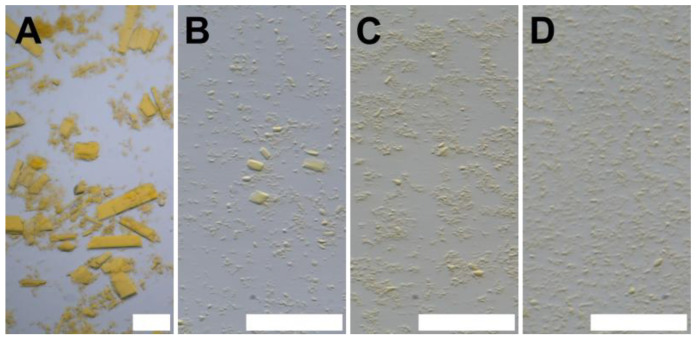
Iq-mEmerald seedstock preparation. A large-scale, homogeneous seedstock was obtained after vortexing a mixture of different-sized crystals with glass beads. (**A**) Inhomogeneous batch crystal suspension; (**B**) after 5 min of vortexing; (**C**) after 10 min of vortexing; (**D**) after 20 min of vortexing. Scale bars: 50 μm.

A large seedstock was produced from an initially inhomogeneous batch crystallization setup. Several rounds of vortexing were needed to obtain a suitable seedstock (Figure 3). Only after vigorous vortexing for 20 min, a seedstock, not containing larger crystal fragments, was produced. Seedstocks should be prepared in sufficient volumes so that a single batch can be used for an entire experiment. The seedstocks were stored at room temperature for several months without noticeable degradation.

The size and morphology of the crystals influence mixing dynamics and diffusion time scales. Iq-mEmerald crystal slurries can be reproducibly prepared in various morphologies, including needles, rectangular nuggets, and cubes, with various sizes, making them an ideal standard sample for applications such as nozzle design and jetting tests.

**Figure 4:**
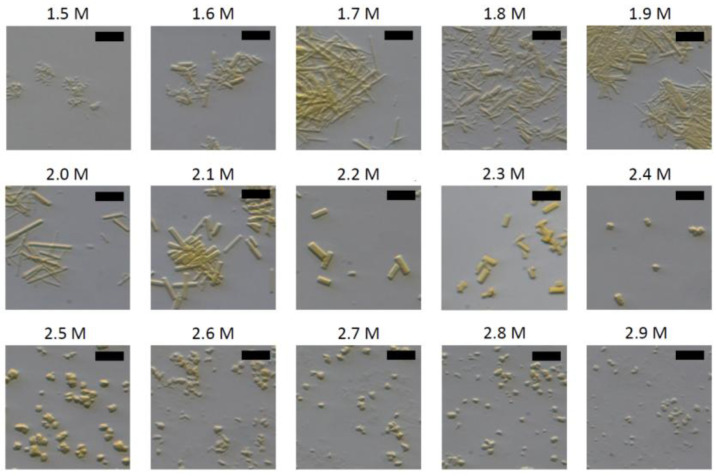
Iq-mEmerald crystal morphology is dependent on the (NH_4_)_2_SO_4_ concentration. The depicted values above the respective images relate to the respective precipitant solutions, not the final (NH_4_)_2_SO_4_ concentrations of the crystallization setups. Scale bars: 10 µm.

For iq-mEmerald, a lower (NH_4_)_2_SO_4_ concentration leads to the formation of needle crystals. The process is independent of adding seedstock. The transition of the crystal morphology happens between the addition of 2 M and 2.5 M (NH_4_)_2_SO_4_ crystallization solutions (Figure 4).

The size of cubic crystals can be adjusted by varying the concentration of (NH_4_)_2_SO_4_ without altering the protein concentration. The crystals mature overnight and remain stable at ambient temperature over extended time periods.

Fluorescence quenching in iq-mEmerald crystals was observed upon mixing them using a mix-and-inject nozzle [63]. To quench the fluorescence of iq-mEmerald microcrystals, a 20 mM CuCl_2_ (in 50 mM Tris, pH 8.0) solution can be used. The quenching can be used to study different nozzles or the diffusion time in various media using a high-speed camera. For the analysis of a mixing HVE nozzle, iq-mEmerald needle crystals were embedded in LCP and the injection was stable with a clear fluorescence signal from crystals (Figure S1E). A 20 mM CuCl_2_ solution, also embedded in LCP, was used to assess successful mixing using the mixer at different flow rates/mixing times.

For the injection of the sample using a DFFN, 15% (*v/v*) cell pellet in the crystallization solution was used and a stable jet was produced (Figure S1D), leading to a reasonable hit-rate (not published). The flexibility of crystallization morphologies and the ability of fluorescence quenching in crystals make iq-mEmerald a potentially useful new standard sample for various applications.

### 3.4. PYP

Photoactive Yellow protein (PYP) from *Ectothiorhodospira halophila* is a well-studied photoactive protein used in many early time-resolved experiments using pump-probe approaches [66,73,112,113]. PYP is dynamic, showing a reversible photocycle in crystals, and does not need to be produced or handled in the dark. This makes it an ideal standard sample for SFX experiments.

Our protocol is on the basis of Schmidt et al. (2019) and Kim et al. (2013) [105,106] but with modifications, leading to higher quantities (over 50 mg/L from 1 L cell culture) as reported before [114,115]. We also adapted the production of the chromophore (*p*CA) from Thomson et al. (2019) [116] and Kim et al. (2013) [106] to streamline the production and purification time to two days (excluding expression).

For crystallization, the use of seedstock leads to the rapid production of large quantities of highly homogeneous microcrystals, usually within a few hours.

#### 3.4.1. Upscaling of Protein Production

As huge amounts of protein are needed for a conclusive time-resolved experiment with several time-points, a robust, high-efficiency expression and purification protocol is important for standard samples. As the purification of PYP is rather cumbersome, it was optimized where possible.

**Figure 5:**
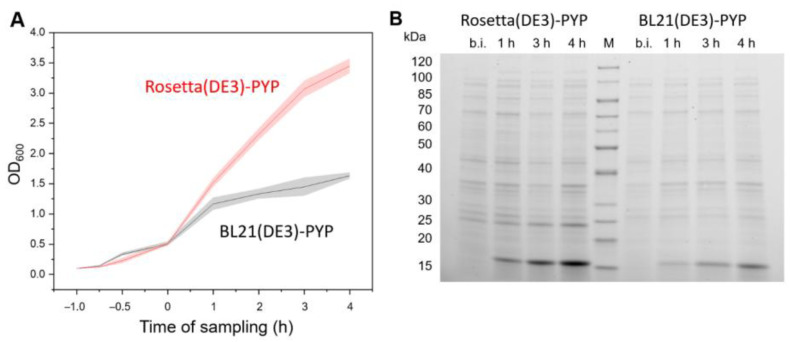
Recombinant expression of *pyp *in different *E. coli* expression strains. (**A**) Growth curves of Rosetta(DE3)-PYP and BL21(DE3)-PYP. For each strain, two single colonies were used to inoculate a pre-culture each. From each pre-culture, three main cultures were inoculated, respectively, with a start OD_600_ of 0.1. The OD_600_ was measured 60 min and 30 min before the induction, as well as 1 h, 2 h, 3 h and 4 h after induction with 1 mM IPTG. The lines depict the OD_600_ averages for each expression strain, whereas the standard deviations are given in the shaded areas. (**B**) SDS-PAGE comparison of recombinant PYP (~14 kDa) expression. M: Marker, 1 h, etc., depict the time of sampling after induction with 1 mM IPTG, b.i.: before induction with IPTG. The raw SDS-PAGE image is provided in Figure S2.

The growth curves of Rosetta(DE3)-PYP and BL21(DE3)-PYP revealed that after induction with IPTG, the growth of BL21(DE3)-PYP was slowed down, leading to lower cell volumes in comparison with Rosetta(DE3)-PYP (Figure 5A). Higher gene expression values in Rosetta(DE3)-PYP cells were also visible on SDS-PAGE analysis (Figure 5B). Hence, Rosetta(DE3)-PYP was used as the expression host.

#### 3.4.2. Seedstock Preparation and Crystallization

To make the crystallization quick, efficient and robust, seedstock was produced, as our initial crystallization trials led to inhomogeneous crystal slurries.

**Figure 6:**
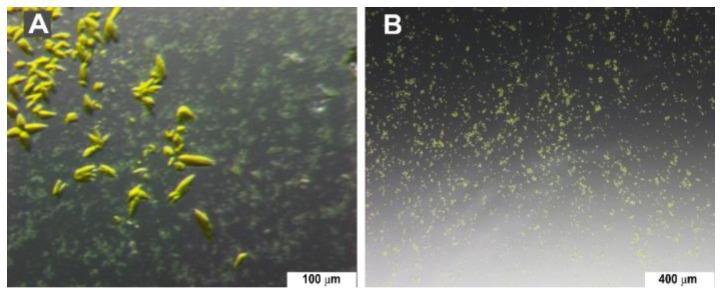
PYP crystals. (**A**) Inhomogeneous large crystals were obtained and used as a starting suspension for the preparation of seedstock. (**B**) Homogeneous PYP microcrystals obtained using seedstock.

A large volume of seedstock was produced from an inhomogeneous batch crystallization setup. Several rounds of vortexing were needed to achieve a suitable seedstock (Figure 6).

Using different seedstock volumes, we could modulate the size of the crystals and crystals were ready in 1 h after crystallization was setup, allowing modifications of crystal size even during an ongoing experiment.

The crystals float on top of the solution due to the higher density of the crystallization solution, making it difficult to accurately estimate the crystal density. To generate a stable liquid jet, the sample volume was adjusted to 1.5 times compared to the total volume used in crystallization (Figure S1F).

The optimized expression and purification protocol, as well as the crystallization using seedstock, led to a reliable and reproducible production of PYP microcrystals in large amounts, making them a useful standard sample for pump-probe experiments.

## 4. Discussion

In this review, we present detailed and reproducible protocols for the preparation and crystallization of lysozyme, myoglobin, iq-mEmerald, and PYP, specifically adapted for SFX. These standard samples were chosen for their straightforward crystallization properties, well-characterized structural data, and relevance to a wide range of SFX applications, including time-resolved studies and instrument commissioning.

Each protein system required specific modifications to optimize crystal quality and to make suitable sample delivery methods. Lysozyme, as a widely used benchmark sample, was crystallized under controlled conditions to ensure consistent size and density. The size of the crystals can be easily modulated by using different temperatures, without the need to change the concentration of precipitant solutions or Lysozyme. Myoglobin crystallization was refined by precise adjustment of precipitant concentrations and, where necessary, deoxygenation steps to support studies of redox-state and ligand binding. The iq-mEmerald system, with its intrinsic fluorescence, provided real-time monitoring of sample injections and diffusion in mixing nozzles as well as the ability to easily produce crystals with different sizes and morphologies. Lastly, we provided a PYP preparation protocol of improved yield and shortened purification and crystallization times. The crystallization of PYP required using a seedstock, which significantly improved crystal homogeneity and growth reproducibility.

All four standard samples, lysozyme, myoglobin, iq-mEmerald, and PYP, exhibit stable crystal suspensions for a minimum of six months when stored at room temperature. To ensure optimal sample quality prior to use, an additional filtration step is performed immediately before injection to remove any large particles or aggregates that may have formed during storage. This approach maintains consistent crystal size distribution and preserves sample homogeneity, critical for reliable serial crystallography experiments. This long-term also benefits their use as convenient and reliable standard samples for serial crystallography experiments.

Each of these protein crystal samples has been validated through data collection at the SPB/SFX instrument at the European XFEL. Under standard experimental conditions, lysozyme, myoglobin, and iq-mEmerald crystals diffracted to approximately 1.7 Å, 1.6 Å, and 1.8 Å resolution at 9.3 keV, respectively, while PYP crystals reached 1.3 Å at 11.56 keV. These high-quality diffraction results confirm the suitability of these proteins as standard samples for serial crystallography. It is noted that even higher resolutions may be attainable, as the current limits were set by the used photon energy and detector distance rather than intrinsic crystal quality. Together, these outcomes demonstrate both the practical reliability and benchmarking value of the selected standards for SFX applications at XFEL facilities.

The standardized methods presented here not only facilitate accurate and reproducible data collection but also support the broader adoption of SFX by lowering technical barriers for new users and laboratories. By providing clear, stepwise protocols, this work contributes to the harmonization of sample preparation practices across the SFX community, enabling more consistent benchmarking and comparison of experimental results.

While our focus remains on lysozyme, myoglobin, iq-mEmerald, and PYP, we also highlight other proteins and biological particles, such as thermolysin, glucose isomerase, proteinase K, trypsin, other GFP derivatives, thaumatin, and granulovirus that serve as alternative or complementary standards for SFX. These additional samples offer unique properties and extend the versatility of SFX for a wider range of structural biology challenges.

## 5. Conclusions

The protocols presented in this review provide a reliable framework for preparing high-quality standard protein crystals suitable for SFX experiments. The standardized methods contribute to the advancement of structural biology by enabling accurate and reproducible data collection and facilitating proof-of-concept studies as well as instrument commissioning. By streamlining such applications, these processes accelerate the generation of biologically meaningful structural insights and contribute to a deeper understanding of biomolecular structure and dynamics.

## Supplementary Materials

The following supporting information can be downloaded at https://www.mdpi.com/article/10.3390/biom15111488/s1.mdpi.com/article/doi/s1. Figure S1: Sample injection images; Figure S2: The unprocessed SDS-PAGE image used in Figure 5. References [117,118] are cited in the Supplementary Materials.
