# Supplementary material for: Standard Sample Preparation for Serial Femtosecond Crystallography
Source: Biomolecules. 2025 Oct 22;15(11):1488. doi: 10.3390/biom15111488 (PMC12649929; doi:10.3390/biom15111488)
Supplement: Supplementary file 1 [file biomolecules-15-01488-s001.zip › biomolecules-3896788-supplementary.pdf]

SUPPLEMENTARY MATERIAL

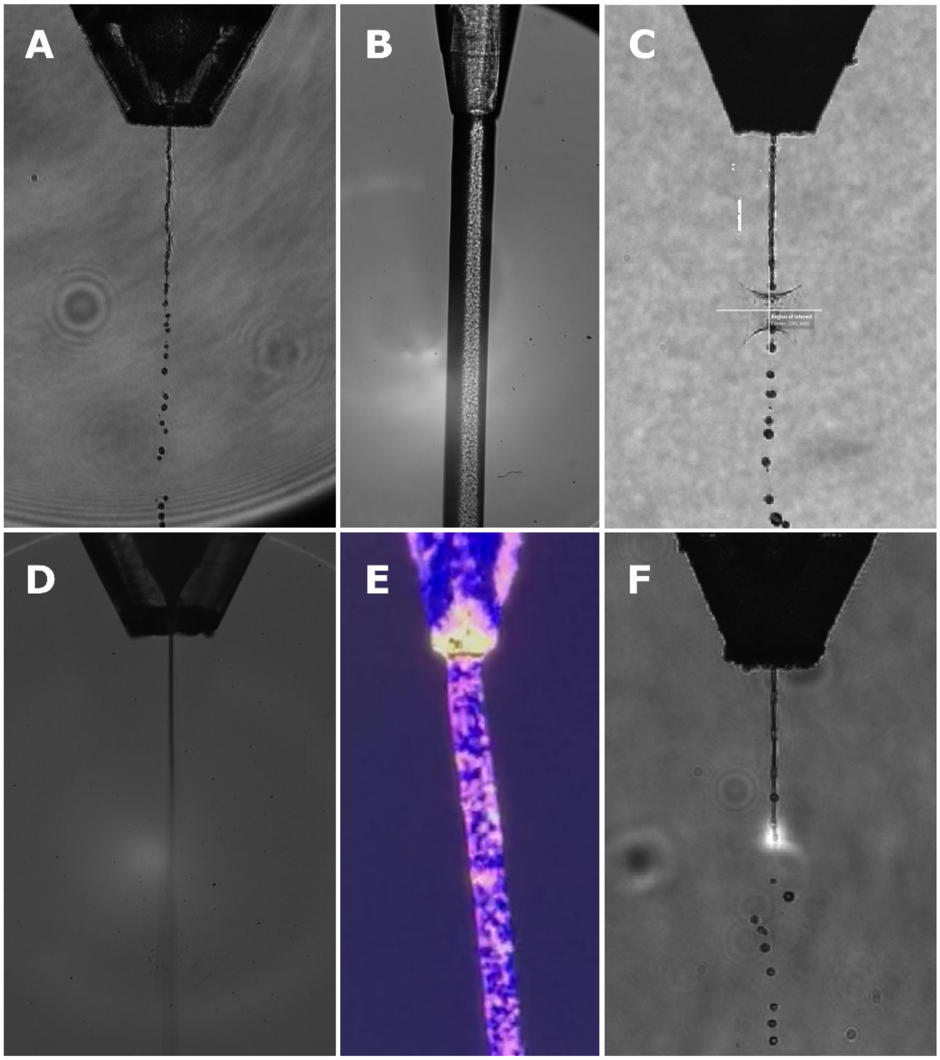

Figure S1. Sample injection images. A) DFFN injection of lysozyme crystals, B) HVE injection of lysozyme crystals embedded in LCP, C) GDVN injection of myoglobin crystals, D) DFFN injection of iq-mEmerald crystals, E) HVE injection of iq-mEmerald crystals embedded in LCP, F) GDVN injection of PYP crystals. A), C), and F) were taken in SPB/SFX instrument sample chamber with nanosecond illumination [118]. B), D), and E) were taken at sample delivery test station in the lab [119]. E) was imaged with fluorescence [63].

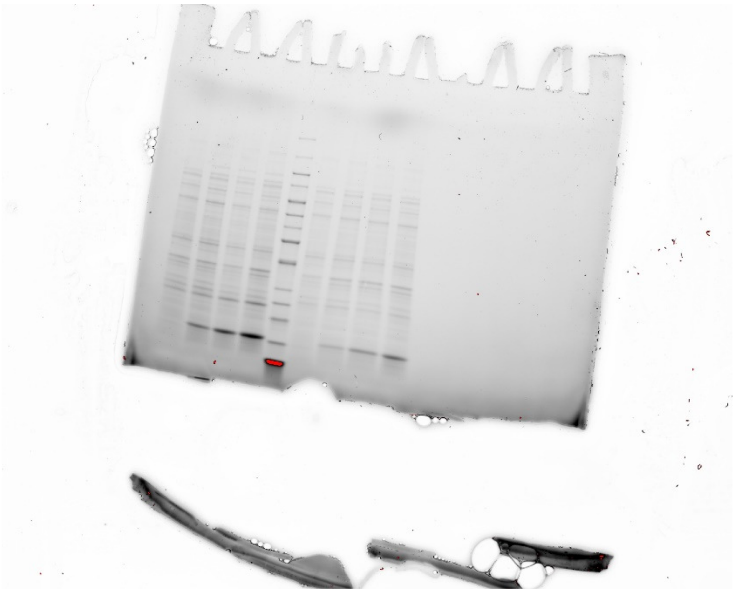

Figure S2. The unprocessed SDS-PAGE image used in Figure 5
